# Fusion of fibrous cap thickness and wall shear stress to assess plaque vulnerability in coronary arteries: a pilot study

**DOI:** 10.1007/s11548-016-1422-3

**Published:** 2016-05-28

**Authors:** Guillaume Zahnd, Jelle Schrauwen, Antonios Karanasos, Evelyn Regar, Wiro Niessen, Theo van Walsum, Frank Gijsen

**Affiliations:** 1 Biomedical Imaging Group Rotterdam, Department of Radiology & Nuclear Medicine and Department of Medical Informatics, Erasmus MC, Rotterdam, The Netherlands; 2 Department of Biomedical Engineering, Thorax Center, Erasmus MC, Rotterdam, The Netherlands; 3 Department of Interventional Cardiology, Thorax Center, Erasmus MC, Rotterdam, The Netherlands

**Keywords:** Fibrous cap thickness, Wall shear stress, Optical coherence tomography, Angiography, Coronary artery, Atherosclerotic plaque

## Abstract

**Purpose:**

Identification of rupture-prone plaques in coronary arteries is a major clinical challenge. Fibrous cap thickness and wall shear stress are two relevant image-based risk factors, but these two parameters are generally computed and analyzed separately. Accordingly, combining these two parameters can potentially improve the identification of at-risk regions. Therefore, the purpose of this study is to investigate the feasibility of the fusion of wall shear stress and fibrous cap thickness of coronary arteries in patient data.

**Methods:**

Fourteen patients were included in this pilot study. Imaging of the coronary arteries was performed with optical coherence tomography and with angiography. Fibrous cap thickness was automatically quantified from optical coherence tomography pullbacks using a contour segmentation approach based on fast marching. Wall shear stress was computed by applying computational fluid dynamics on the 3D volume reconstructed from two angiograms. The two parameters then were co-registered using anatomical landmarks such as side branches.

**Results:**

The two image modalities were successfully co-registered, with a mean (±SD) error corresponding to $8.6\,\pm \,6.7\,\%$ of the length of the analyzed region. For all the analyzed participants, the average thinnest portion of each fibrous cap was $129\,\pm \,69\,\upmu \text {m}$, and the average WSS value at the location of the fibrous cap was $1.46\,\pm \,1.16\,\text {Pa}$. A unique index was finally generated for each patient via the fusion of fibrous cap thickness and wall shear stress measurements, to translate all the measured parameters into a single risk map.

**Conclusion:**

The introduced risk map integrates two complementary parameters and has potential to provide valuable information about plaque vulnerability.

## Introduction

Cardiovascular disease is the leading cause of mortality and morbidity worldwide [43]. Acute myocardial infarction is generally triggered by rupture of so-called vulnerable atherosclerotic plaques in the coronary artery. Such rupture-prone plaques are often referred to as “silent killers,” since symptoms remain unnoticed until the event. Therefore, identifying vulnerable plaques to determine whether and where to stent is of eminent importance and represents a major clinical challenge.

The morphological characteristics of rupture-prone plaques are a large lipid necrotic core, an overlying thin fibrous cap, and dense macrophage infiltration [27]. These vulnerable plaques are also known as thin-cap fibroatheromas and are considered the precursor phenotype of plaque rupture [39]. Fibrous cap thickness is the most critical component of plaque stability, namely thinner caps being more prone to rupture than thicker caps. The threshold of $151\,\upmu \text {m}$ was demonstrated to be the best cutoff to predict rupture for most representative fibrous caps [45]. Therefore, in vivo quantification of fibrous cap thickness could enable identification of vulnerable plaques and potentially guide appropriate treatment such as percutaneous coronary intervention prior to the occurrence of an event.

Complementary parameters to assess plaque vulnerability can be derived from information about local hemodynamics. Wall shear stress (WSS) is the frictional force of the blood at the vessel wall and plays an important role in the development and progression of atherosclerotic plaques [31, 41]. In carotid artery disease, evidence that WSS plays an important role in plaque destabilization is compelling. Not only is the lipid core larger and the cap thinner in the upstream high WSS region [4, 7, 44], the location of plaque rupture is also associated with increased WSS levels [4, 7, 32]. Although studies in coronary arteries were often smaller in size than the studies dealing with carotid plaques, similar findings were reported correlating WSS levels with the location of cap-thinning [13], the growth of the necrotic core [22, 42], and the location of the rupture [9, 11]. Accordingly, combining WSS computations with cap thickness measurements would constitute a new risk index that potentially improves the identification of vulnerable plaques in coronary arteries.

Fibrous cap thickness can be quantified accurately in vivo with intravascular optical coherence tomography (OCT) [17]. OCT is a catheter-based imaging modality that enables tissues to be visualized in vivo at a high spatial resolution (10–20 $\upmu$m). Investigation of the inner circumference of the vessel is performed by the probe spinning along its axis while being pulled back. The emission and reception of near-infrared light at each angular step yield the acquisition of so-called *A*-lines, whose echo time and intensity are then converted into a single grayscale image. During the pullback acquisition, a stack of consecutive cross-sectional images is generated along the length of the assessed artery segment. The very high spatial resolution of OCT enables an accurate characterization of the structure of the most superficial layers of the arterial wall. The near-histology resolution of OCT can indicate the degree of subclinical atherosclerotic lesion formation and be used to quantify accurately fibrous cap thickness [33].

WSS can be derived from computational fluid dynamics (CFD), namely by computing the arterial blood flow. Endothelial WSS is quantified by calculating the derivative of the computed flow field at the surface of the wall. To perform such computation, the three-dimensional (3D) arterial lumen geometry is required. Previous work showed that 3D arterial geometry can be accurately reconstructed from two angiography images (i.e., at two different angles) [14, 36]. Although this procedure involves the assumption that the luminal contour can be approximated to an elliptical shape, the impact of this approximation is minimal in mildly diseased arteries [34], as it is the case in the present study. Such reconstructions have been successfully used to compute WSS in coronary arteries in several recent studies [15, 16].

Co-registration of several imaging modalities to improve plaque analysis has been investigated in previous studies. A method for accurate side branch modeling using angiography and OCT was proposed in a recent work [18]. In another study [34], WSS was computed from volumetric reconstructions generated from 3D OCT and compared with corresponding geometries derived from 3D intravascular ultrasound (IVUS) and 3D angiography. The effect of inflow boundary conditions on WSS was also investigated using IVUS and angiography [20]. The relationship between WSS and plaque characteristics was assessed using a different approach [37] with OCT and angiography. Nevertheless, the fusion of WSS and cap thickness has not been addressed yet, and the clinical relevance of the resulting index corresponding to the fusion of these two parameters has not been investigated.

The aim of the present study is to propose a novel tool devised to evaluate the risk of plaque rupture in clinical settings, by exploiting the fusion of in vivo OCT and angiography imaging. A methodology is proposed for a combined assessment of fibrous cap thickness and WSS information. Fibrous cap thickness is quantified from OCT images using a previously validated method that was developed in-house [46]. WSS is computed from angiograms by exploiting a recently proposed method [15]. A co-registration framework devised to accurately align and fuse the two measured parameters is presented. Although OCT is a real-time imaging modality, and current techniques enable WSS computations to be performed relatively quickly (i.e., within a few minutes), co-registration of these two parameters remains quite challenging to perform online. In the present feasibility study, all computations are therefore performed off-line, as the main contribution of this work is the introduction of a unique index that is derived from co-registered parameters and displayed as a single risk map aiming to improve the identification of high-risk regions in coronary arteries. To evaluate the feasibility of the proposed framework, a proof-of-concept validation is carried out in 14 patients.

## Methods

### Study population

Data were gathered at the Thoraxcenter, Erasmus MC (Rotterdam, The Netherlands). Fourteen patients (mean age $61.0\,\pm \,10.3$ years old, 7 males), suffering from coronary artery disease and referred for possible percutaneous coronary intervention, were involved in the study. The most representative and largest plaque was selected in each pullback. The only inclusion criteria were the presence of a fibrous plaque (nine patients) or a fibrocalcific plaque (five patients) in the acquired pullbacks. The image selection was reviewed by a clinician expert in OCT imaging. Ten left anterior descending arteries, one left main artery, and three right coronary arteries were simultaneously imaged with angiography and OCT. Informed consent was acquired from the patients for use of their imaging data. All procedures followed were in accordance with the ethical standards of the responsible committee on human experimentation (institutional and national) and with the Helsinki Declaration of 1975, as revised in 2008 (5).

### Data acquisition

Angiography recordings were acquired at a frame rate of 15 frames/s with the Axiom Artis system (Siemens, Forchheim, Germany). The two recording angles were selected to avoid overlap of branches other than the selected coronary artery. Table movement in between the two recordings was avoided as well. The ECG signal was registered during the recordings. The angiography images were acquired at $1024 \times 1024$ pixels, with a pixel size of $76\,\upmu \text {m}$.

OCT imaging was realized with three different scanners, to assess the applicability of the framework. Pullbacks were acquired using one of the following apparatus: (1) Ilumien frequency domain imaging system with the Dragonfly Duo intracoronary imaging catheter (Lightlab/St Jude, Minneapolis, MN, USA) (nine patients), (2) Lunawave system with the Fastview imaging catheter (Terumo Corporation, Tokyo, Japan) (two patients), or (3) MGH prototype optical frequency domain imaging system (Massachusetts General Hospital, Boston, MA, USA), and the Terumo Fastview catheter (three patients). Image acquisition was performed with a previously described non-occlusive technique [33]. For the Lightlab system, pullbacks were acquired over a total length of either 54 mm (six patients) or 75 mm (three patients) along the vessel, at either 105 or 180 frames/s, with an inter-frame distance of either 200 or 100 $\upmu$m. For the Terumo system, pullbacks were acquired over a total length of 47 mm, at 160 frames/s, with an inter-frame distance of 125 $\upmu$m. For the MGH system, pullbacks were acquired over a total length of 61, 81, and 121 mm along the vessel, at 100 frames/s, with an inter-frame distance of 200 $\upmu$m. For all scanners, the spatial resolution was 20 and 30 $\upmu$m in the axial and lateral directions, respectively. The depth of the scan range was 4.3 mm. Acquired images were sampled at $968 \times 504$ pixels.

### Quantification of fibrous cap thickness from OCT images

Fibrous cap thickness was quantified using a previously validated framework [46, 47]. This semi-automated approach consists in a robust contour segmentation scheme based on the fast marching methodology [5]. Briefly, a cost function is derived from the intensity gradient of the polar OCT image along the radial direction. A front propagation scheme is then run, favoring low cost points (i.e., data attachment term) while penalizing radial displacements (i.e., smoothness constraint term). The optimal path, corresponding to the segmentation contour, is finally extracted by means of a back-tracking scheme in the propagated values.

The three principal phases of the framework are (i) a manual selection of the region of interest (ROI) containing the fibrous cap to be analyzed; (ii) the automatic extraction of the luminal interface over the entire vessel circumference; and (iii) the automatic extraction of the abluminal interface of the fibrous cap within the ROI. Fibrous cap thickness is finally calculated as the distance between both contours of the cap, along a set of lines perpendicular to the luminal interface.

### Computation of wall shear stress from angiography images

To compute WSS, the lumen of a coronary artery was first reconstructed based on two angiography images, following a previously adopted method [15]. These two angiography images were recorded with angular difference of at least $30^{\circ }$ and were selected in the same cardiac phase, as determined from the ECG information. The contrast-filled lumen in the two-dimensional (2D) images was segmented by a trained observer (JS). From the 2D segmented contours, a 3D volume was generated, using validated commercially available software (CAAS v5.11, Pie Medical Imaging, Maastricht, the Netherlands) [14]. Extension at the inlet and the outlets of five times the radius was added to exclude computations artifacts [29]. Next, based on the volume of the lumen, a mesh was generated to compute WSS.

The volume meshes were built using the standard meshing tools in ICEM (ANSYS ICEM-CFD v14.5, Ansys Inc, Canonsburg, USA). Prior to the final simulation, a grid dependency study was performed. The grid size was decreased stepwise until the velocity and wall shear stress did not differ more than $3\,\%$ for each node. This resulted in a typical cell size of 0.1 mm. At critical points (i.e., stenoses and curvature in bifurcation regions), the cell size was reduced up to $30\,\%$ of the original size. A five-element layer of prism cells was added to optimally capture boundary layer effects. Finally, this resulted in a typical mesh size of $2\times 10^6$ cells. The inlet and outlet boundary conditions for these computations were assigned by applying scaling laws derived in a previous study [10]. In that study, the absolute inflow and the outflow ratio over bifurcations were derived from in vivo measurements in mildly diseased coronary bifurcations. The measured flow rate was subsequently related to the local diameter of the mother and daughter branches. The boundary conditions for our study were assigned by applying these diameter-based scaling laws. WSS was calculated with CFD using a finite volume solver and by applying standard numerical techniques to perform steady-state computations (Fluent v14.5, Ansys Inc, Canonsburg, USA) [28].

From the results, the 3D WSS magnitude at the wall was extracted and converted to a 2D coordinate system. To do so, the planes perpendicular to the centerline were defined at 0.2 mm intervals. The planes were subsequently subdivided into intervals of $10^\circ$. The average WSS values within the resulting bins were mapped to a 2D representation.

We recently demonstrated that normalized WSS is especially robust for potential errors in inflow and outflow boundary conditions [28]. Therefore, in addition to absolute WSS, this parameter was investigated as well. For the normalization procedure, each 2D WSS map was normalized by its 50th percentile value.

### Co-registration procedure of OCT with angiography

**Fig. 1:**
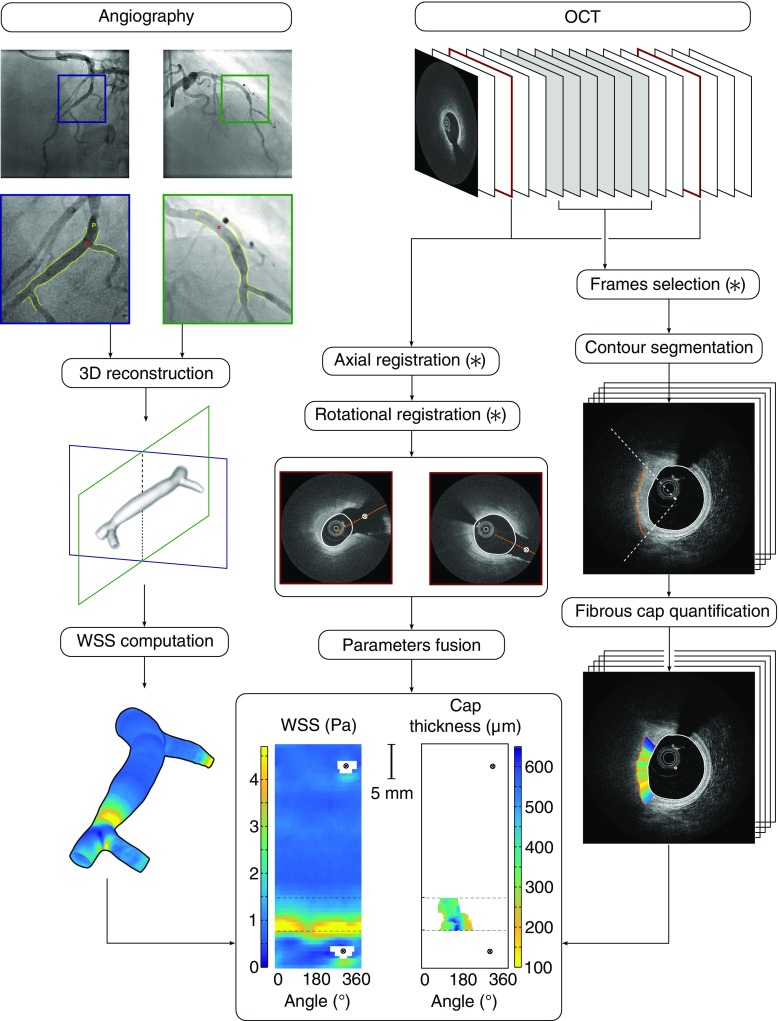
General framework of the method. All steps of the method are (semi-) automatic, except those indicated with an *asterisk* ($*$)

Fusion of OCT and angiography was performed manually using a previously proposed approach [38]. The matching procedure involves two main steps, namely axial and rotational registration (Fig. 1). Axial registration consists in aligning the length and position of a given arterial segment of the OCT pullback with the corresponding segment in the 3D reconstructed vessel. Rotational registration is the task to determine the angular transformation so that the two segments have the same orientation and overlap with each other. These two steps are described below.

#### Axial registration

The OCT catheter was visible in at least one of the two angiography images and was used to localize the starting and ending points of the OCT pullback. Additional landmarks such as bifurcations and narrowings were identified in both OCT and angiography images. To correctly identify the landmarks, their size as well as the distance between them was also used as an additional information. Registration was performed by matching similar pairs of landmarks between the two imaging modalities.

#### Rotational registration

Due to the vessel tortuosity, one full rotation of the OCT probe during the pullback acquisition may not systematically correspond exactly to $360^{\circ }$. This has very little effect on a single frame, but can introduce an important shift across a series of stacked frames. To determine the angular shift between the OCT images and the 3D volume, the orientation of two side branches was used. First, information related to angles was extracted from the cut-open map corresponding to the WSS derived from the 3D reconstruction. In this map, the position of the side branches was indicated. The corresponding positions were also indicated in the OCT pullback. Next, the OCT frames of the distal and proximal side branches were rotated in order to match the orientation of the corresponding side branches in the WSS map. Finally, all OCT frames in between the two side branches were rotated using a linear interpolation between the two extreme rotation angles.

### Parameters fusion

To visually display the risk index of the interrogated region on a single map, fusion of cap thickness and WSS was finally performed after the co-registration of OCT and angiograms. Recent findings demonstrated that the best cutoff to predict rupture was $151\,\upmu \text {m}$ for most representative fibrous caps [45]. Therefore, this value was used as an upper threshold to identify at-risk regions based on cap thickness information. Similarly, $1.7\,\text {Pa}$ is often used to represent the transition between intermediate and high WSS [31], and this value was selected as a lower threshold to characterize at-risk regions based on WSS information. The different risk levels, determined from fusion of cap thickness and WSS, are presented in Table 1.

The sensitivity of both thresholds parameters was assessed by iteratively building nine different risk maps, corresponding to the combination of three different cap thickness thresholds and three different WSS thresholds. The assessed parameter values were equal to the initially determined central value, as well as the central value ${\pm }7\,\%$, namely 141, 151, and 161 $\upmu \text {m}$ for the cap thickness, and 1.6, 1.7, and 1.8 Pa for the WSS. Then, the area of each of the four regions corresponding to a specific risk level (Table 1) was quantified, and the total variability of each region under the nine different parameters settings was calculated.

## Results

### Co-registration of OCT and angiography

For each included patient, at least one site corresponding to an atherosclerotic plaque covered by a fibrous cap could be identified in the OCT pullback. When more than one site was found, the one corresponding to the largest plaque, both axially and circumferentially, was selected. The lesion was subsequently localized in the two corresponding angiograms. Volumetric reconstruction from angiography could not be performed for two cases, due to insufficient image quality of the side branches that were used as landmarks (i.e., presence of a trifurcation with vessels overlapping with each others in the image plane for the first case, and contours poorly visible with high level of noise for the second case). These two patients were removed from the study, and the framework was applied on the 12 remaining cases.

Volumetric 3D geometries were reconstructed with two angiography views corresponding to the same cardiac cycle, based on the recorded ECG. Ten cases were reconstructed in diastole. The two remaining cases were reconstructed in systole, since over-projection of the side branches did not allow the diastolic phase. Nevertheless, only rigid transformation without deformation of the ROI was observed by the trained reader (JS) between the diastolic and systolic phases.

**Table 1:** Risk index

Risk	Cap thickness ($\upmu \text {m}$)	Wall shear stress (Pa)
Low	${>}151$	${<}1.7$
Medium	${>}151$	${\ge }1.7$
Medium	${\le }151$	${<}1.7$
High	${\le }151$	${\ge }1.7$

**Fig. 2:**
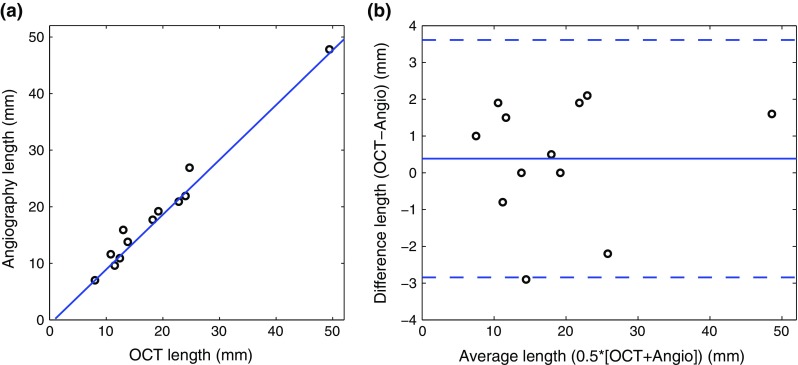
Length validation between the proximal and distal side branches used for the co-registration between OCT and angiography, for the 12 processed cases. **a** Linear regression line and **b** Bland–Altman plot

Cap thickness and WSS were computed using the methods detailed in “Quantification of fibrous cap thickness from OCT images” and “Computation of wall shear stress from angiography images” sections. Co-registration between OCT and angiography was then successfully performed for all cases. Results displaying the co-registration of WSS and cap thickness are presented in Fig. 4, and identification of at-risk regions was finally realized via the fusion of these two parameters in a single map (“Parameters fusion” section).

To quantify the accuracy of the co-registration procedure, the length between the proximal and distal side branches that were used as landmarks (Fig. 1) was measured along the vessel centerline, in both the angiography-derived 3D volume and the OCT pullback. The average length (${\pm }$SD) between the two side branches was $19.0\,\pm \,11.1\,\text {mm}$ in OCT, and $18.6\,\pm \,10.8\,\text {mm}$ in angiography. Regression analysis demonstrated a strong linear relationship between the length derived from the two modalities with $R=0.99$, as depicted in Fig. 2. The mean absolute difference between the distance measured in OCT and angiography was $1.4\,\pm \,0.9\,\text {mm}$, corresponding to $8.6\,\pm \,6.7\,\%$ of the measured length, with a bias of $0.4\,\text {mm}$ and a $95\,\%$ CI of $[-2.8, 3.6]\,\text {mm}$. The average absolute rotation angle between the two side branches was $77^\circ \,\pm \, 53^\circ$, with a rotation angle per frame corresponding to $0.6^\circ \,\pm \,0.4^\circ$.

**Fig. 3:**
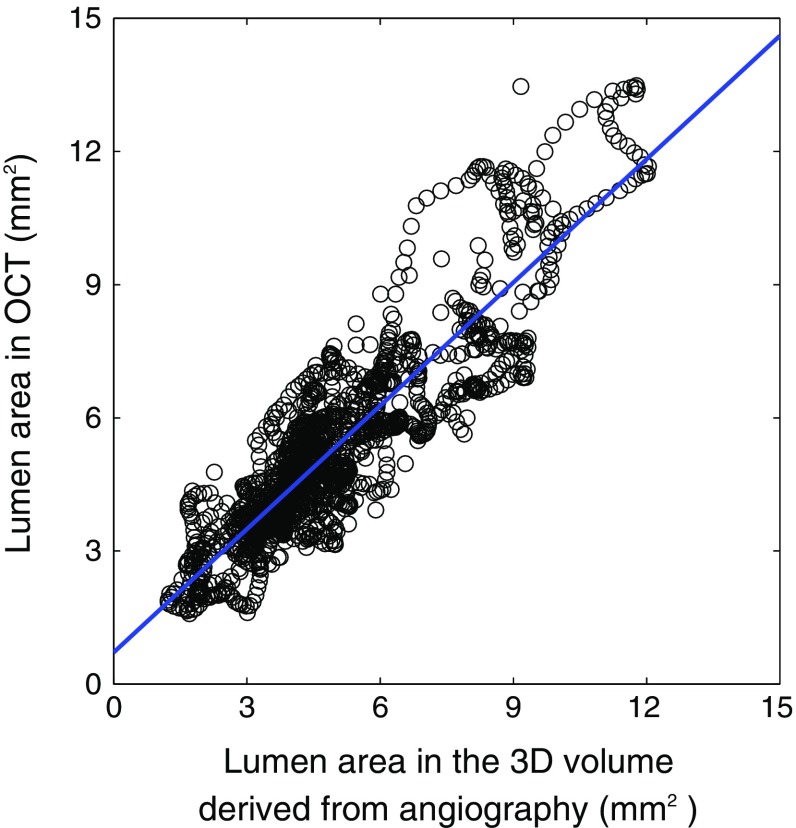
Linear regression line between the lumen area derived from angiography and from OCT, for all 12 processed cases

Additionally, for the 12 analyzed patients, the lumen area of the arterial segment was measured in each OCT frame between the two landmark side branches and compared with the lumen area measured at the corresponding location in the 3D volume derived from angiography. A good correlation ($R=0.89$) was found between the two set of measurements, as displayed in Fig. 3.

### Fibrous cap thickness and WSS analysis

For the 12 analyzed patients, the average (±SD) cap thickness over the entire plaque was $278\,\pm \,119\,\upmu \text {m}$, and the thinnest portion of each fibrous cap was $129\,\pm \,69\,\upmu \text {m}$. The most representative cap thickness value was also quantified, following a previously established approach [45]. Briefly, this value was defined as the average of a collection of cap thickness measurements, performed in the cross-sectional image at five randomly selected sites, and every 0.5 mm along pullback direction throughout the plaque. The average value of the most representative cap thickness for the 12 cases was $280\,\pm \,126\,\upmu \text {m}$. No patients had a representative cap thickness inferior to the $151\,\upmu \text {m}$ threshold [range $(179{-}518\,\upmu \text {m})$]. Along the *z*-axis of the OCT pullback, the average length of the region encompassing the analyzed fibrous cap was $4.5\,\pm \,2.4\,\text {mm}$, corresponding to an average of $33\,\pm \,19\,\text {frames}$. Along the circumference of the vessel, the average angle of the ROI encompassing the plaque was $140^\circ \,\pm \,58^\circ$.

The average WSS value at the location of the fibrous cap was $1.46\,\pm \,1.16\,\text {Pa}$. Such magnitude is typically considered as a medium WSS value. However, the normalized WSS value at the location of the fibrous cap was $1.58\,\pm \,1.12$. This operation was performed by normalizing the values with respect to the median WSS, within the entire region located between the two side branches used for the co-registration. The normalized value is greater than 1, which implies that the WSS at the location of the fibrous cap was relatively higher than the WSS values of the surrounding regions. Only one case had a relatively lower WSS at the ROI, with a normalized value of $0.59\,\pm \,0.13$. This demonstrates that, in all cases but one, the highest stress values also corresponded to the location of the fibrous cap.

No direct association between WSS and cap thickness was observed. For the 12 analyzed patients, the average composition of the risk map (Fig. 1) was the following: $35\,\%$ of the total region corresponded to a high WSS (i.e., ${\ge }1.7\,\text {Pa}$, medium risk), $5\,\%$ to a thin cap (i.e., ${\le }151\,\upmu \text {m}$, medium risk), $2\,\%$ to both of these two factors combined (i.e., high risk), and $58\,\%$ to none of them (i.e., low risk). For three patients, more than $80\,\%$ of the plaque was under high WSS levels. For two other patients, a thin cap covered more than $20\,\%$ of the total plaque region. For another patient, both of these two factors were present in an area equal to $15\,\%$ of the plaque. The other participants did not meet these criteria and had a plaque with relatively smaller regions of elevated WSS and/or thin fibrous cap.

For the 12 assessed cases, the distance between the center of the analyzed plaque and the two side branches encompassing the ROI was assessed. No caps were present in the bifurcation regions. The average distance was $11\,\pm \,9\,\text {mm}$ from the proximal side branch and $9\,\pm \,6\,\text {mm}$ from the distal side branch. By subdividing the arterial segment between the proximal and distal side branches into four sections of identical length, the plaque was located in the first section for one case, in the second section for three cases, in the third section for four cases, and in the fourth section for four cases.

The sensitivity of both the cap thickness threshold and the WSS threshold was assessed by generating a set of different risk maps with parameter values that ranged between ${\pm }7\,\%$ of the central value. For all cases, the mean total variability of the area of the regions in the risk map was the following: from ${-}7$ to ${+}6\,\%$ for the gray region (low risk); from ${-}9$ to ${+}9\,\%$ for the blue region (intermediate risk); from ${-}31$ to ${+}55\,\%$ for the yellow region (intermediate risk); and from ${-}67$ to ${+}70\,\%$ for the red region (high risk).

### Computation time

The computations were performed on a desktop computer equipped with an Intel Xeon 2.4 GHz processor with 4 cores and 12 GB of memory. For each patient, the average computation time was the following: 7 s to compute the fibrous cap thickness, 10 s to generate the 3D geometry reconstruction, and 2 h to compute the WSS.

## Discussion

In this pilot study, two image-based parameters, fibrous cap thickness and WSS, derived from two different image modalities, OCT and angiography, were assessed in the coronary artery and subsequently co-registered. OCT imaging enables arterial tissues to be visualized in vivo at a near-histology resolution and can detect high-risk morphologies such as thin-cap fibroatheromas. Angiography can provide 3D information about arterial geometry, local hemodynamics, as well as WSS, thus potentially enabling fusion of complementary parameters to assess the rupture risk of a certain plaque. The principal contribution of this work is the introduction of a unique risk map that integrates complementary information derived from cap thickness and WSS parameters.

### Co-registration of fibrous cap thickness and WSS

Proof-of-concept results were obtained by processing 12 coronary segments imaged from 12 in vivo patients. An atherosclerotic plaque covered by a fibrous cap was identified in all OCT pullbacks. In all cases, the computed WSS was within the expected physiological range, with elevated WSS at the plaque location. Co-registration between OCT and angiography was performed using two side branches as anatomical landmarks. The accuracy of the co-registration method was validated by the high agreement between the corresponding lengths of the co-registered segments.

The 12 patients analyzed in this study had a representative fibrous cap of intermediate thickness (i.e., $280\,\pm \,126\,\upmu \text {m}$). Nevertheless, the average value at the thinnest point of the cap was $129\,\pm \,69\,\upmu \text {m}$, which is inferior to the lower threshold of $151\,\upmu \text {m}$ used to characterize at-risk regions. It is expected that a broader range of cap thickness, including caps thinner than $65\,\upmu \text {m}$, would be observed by including additional patients in the study. The method to quantify cap thickness was developed in-house and previously validated [46]. The clinical applicability of the proposed method is supported by a relatively accurate quantification of cap thickness, with a mean absolute error (±SD) of $30\,\pm \,37\,\upmu \text {m}$.

The association between coronary WSS levels and various atherosclerosis markers was investigated in several studies [31, 34, 37]. Nevertheless, these studies do not include the side branches in their 3D reconstruction framework when computing the WSS. Although such approaches simplify the modeling procedure, the presence—or absence—of side branches critically influences the resulting flow phenomena near bifurcations, as demonstrated in a recent study [18]. The computed flow field in a straight segment directly in between two side branches is not likely to result in adequate WSS patterns, especially at the proximal and distal ends. Moreover, bifurcated areas also correspond to predilection sites for atherosclerosis development, thus making accurate computation in these areas particularly pressing. In the present study, special care has been taken to incorporate both proximal and distal side branches in the CFD computation in order to provide reliable WSS levels.

**Fig. 4:**
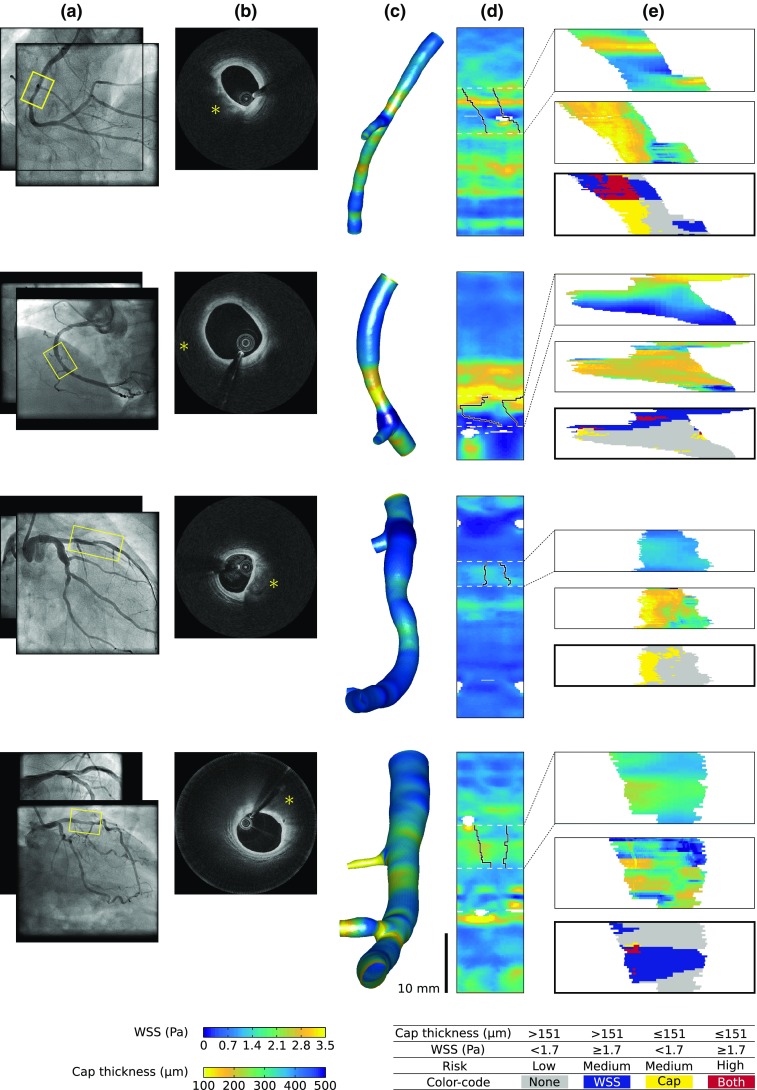
Result examples. **a** Two angiograms. The region of interest (ROI) is indicated with the rectangle. **b** OCT image. The fibrous cap is indicated with the *asterisk*. **c** 3D reconstruction of the ROI. **d** WSS map. The region corresponding the fibrous cap is indicated by the *dashed lines* and the *violet contours*. **e** Fibrous cap regions. *Top row* WSS map (magnified). *Middle row* co-registered cap thickness map. *Bottom row* corresponding risk index. Patient 1: thin fibrous cap (*yellow*) with localized high WSS (*blue*), defining a high-risk region (*red*). Patient 2: low to medium WSS with relatively thick fibrous cap. Patient 3: thin cap with low WSS. Patient 4: medium WSS with relatively thick fibrous cap

A unique and patient-specific risk index map (Fig. 4e) could successfully be generated for all cases. This tool translates all the measured parameters into a single map for interpretation of the results. The proposed risk index map enables fast and simple identification of at-risk lesions. Such approach has potential to provide a useful aid for interventional planning and decision making. In this pilot study, all the (semi-) automatic steps of the framework (Fig. 1) are currently processed off-line. The implementation of the method can be optimized by running the code on a graphics processing unit, thus resulting in faster computations and enabling online utilization of the tool directly in the catheterization laboratory.

### Clinical implications

Both angiography and OCT imaging provide valuable information to evaluate the patient, namely WSS and cap thickness. No clear relationship was found between extreme WSS values and cap thickness; therefore, the two parameters are likely not to be redundant but instead complementary. Thin fibrous cap reflects a short-term risk of plaque rupture, while elevated WSS better indicates a midterm risk. The underlying concept of atherosclerosis is complex, and new approaches are required to better assess the risk of the patient.

In this study, threshold values are used to define the risk index from cap thickness measurements and WSS computations. Regarding fibrous cap thickness, the threshold of $65\,\upmu$m is widely adopted to identify high-risk lesions [2]. However, this empirical value is likely to be underevaluated, since ex vivo tissues usually undergo variable shrinkage rate during histological preparation [33, 39]. Moreover, in such pathology studies [39], vulnerable plaques from patients who died from cardiovascular disease were included. Assuming that these patients harbor a more vulnerable plaque phenotype than our population implies that the cap thickness in our study should be larger as well. A different study established that ruptured plaques in acute coronary syndrome are often associated with a fibrous cap thickness of up to $100\,\upmu$m [35]. Another group [45] reported a value of $80\,\upmu$m for the thinnest fibrous cap, and $188\,\upmu$m micrometer for the representative fibrous caps. Therefore, values reported in this study fall within the range presented in that study, especially given the low number of fibrous caps we investigated. In the present work, the $151\,\upmu$m threshold value was adopted, as it has been demonstrated to be the best cutoff to predict plaque rupture for most representative fibrous caps [45].

As for WSS, a consensus in the literature uniformly associates values lower than 1.0 Pa to low WSS levels [26, 41]. Nevertheless, there is still an ongoing debate about the absolute WSS levels used to categorize the high WSS profiles. A first study [3] showed that pathobiological processes started destabilizing the cap above 1.5 Pa. A different team [26] used a cutoff value of 2.5 Pa and reported that this level is associated with a transition into a phenotype of vulnerable plaque. Another study [42] analyzed regions where the WSS levels were above 4.0 Pa, and observed that these regions corresponded to areas with larger necrotic core and higher plaque burden. In the present work, we adopt the threshold value of 1.7 Pa. The choice of this value of 1.7 Pa was motivated by the fact that this value was proposed as the optimal threshold between moderate and high WSS in the largest cohort study to date that investigated atherosclerotic disease progression and localized WSS values [31]. Nevertheless, critical WSS levels need to be further evaluated to reach a consensus and determine the actual clinically relevant values. The novel risk index introduced in the present study can potentially contribute to re-establish relevant WSS values.

The resulting risk map (Fig. 4) depends on the value of both cap thickness and WSS threshold parameters. A certain variability could be observed when changing one or both of the parameter values. The largest variation was observed in the at-risk region, with changes ranging between ${-}67$ and ${+}70\,\%$ of the area of the initial region. The large amplitude of these variations can be explained by the fact that the actual size of this region was also very small (namely $2\,\%$ of the total plaque region); therefore, even a small change in the area would result in a large relative variation.

The clinical significance of this study is also supported by previous work [37] that combined OCT and angiography imaging, and reported that coronary regions exposed to low WSS were associated with larger lipid burden, thinner fibrous cap, and higher prevalence of thin-cap fibroatheroma. Nevertheless, no association was found between peak WSS and low cap thickness in the present study. However, the number of patients involved in this study is relatively limited. Furthermore, results only represent a snapshot in time from which the current values of cap thickness and WSS can be quantified; therefore, the clinical interpretation is limited. Atherosclerosis is a process that develops over time and follows a complex evolution [23]. The different manifestations of the syndrome on the arterial wall, such as plaque composition, morphological alteration, flow patterns, and WSS, also undergo a progressive variation of which the rate may greatly vary from one case to another. A follow-up study would be required to assess the state of the plaque over time, better characterize the evolution of the atherosclerotic process, and assess the added value of the two combined parameters for clinical decision making. Especially for follow-up studies, normalized WSS might be an attractive marker to investigate. Clinical studies have demonstrated a relationship between this marker and enhanced plaque vulnerability [6, 12, 13], and we recently demonstrated that this marker is relatively insensitive to the imposed boundary conditions [28].

### Limitations

In the present study, the most representative and largest plaque was selected in each pullback, and five fibrocalcific plaques were analyzed in addition to seven fibrous plaques. Therefore, the impact of fibrous cap thickness per se was not systematically assessed. Nonetheless, the principal aim of this study was to demonstrate the feasibility of image co-registration between OCT and angiography; therefore, these patients with fibrocalcific plaques were included as well. The 3D volume was generated using a pair of angiography images at two different angles. In such procedure, the cross-sectional lumen geometry was approximated to an elliptic shape. Nevertheless, this assumption had a limited effect on the lumen area, as indicated by the good correlation between the actual lumen area quantified in OCT and the approximated measurements derived from angiography ($R=0.89$, Fig. 3). This is in good agreement with previously reported values [25, 30, 36]. Furthermore, several other studies have shown that angiography-based reconstructions of coronary arteries can be used to provide a good representation of the WSS patterns [15, 29, 34].

Other investigators concluded that WSS from steady-state simulations is in close agreement with time-averaged WSS results from transient computations [8, 16, 21]. Therefore, steady-state computations were performed in this study in order to speed up computation time and make online application feasible. However, this omits the possibility of performing fluid–structure interactions to inspect wall stresses. Future studies, where direct clinical feasibility is less pressing, should focus on correlating wall stresses and rupture risk.

In the present study, 3D reconstruction of the lumen geometry was performed using two angiograms. Therefore, the luminal contour was approximated to an elliptical shape and then used to compute the WSS. Such approximation could result in significant errors (i.e., overly smoothed shear stress maps) when analyzing severely diseased vessels. In these cases, accurate geometry using invasive imaging such as OCT-derived reconstructions would provide more reliable results [25]. Nevertheless, the scope of the present study concerns mildly diseased (i.e., yet untreated) arteries. In such cases, a good agreement was demonstrated between shear maps derived from angiography and OCT [34].

In the scope of the present work, WSS computation took approximately 2 h. In this study, we focus on relevant clinical markers extracted from off-line WSS computations. Nevertheless, optimizing the implementation would enable to decrease the processing time down to a couple of minutes. Therefore, by performing angiography imaging first, subsequent WSS computations could be performed, while the patient is imaged with OCT, and the two imaging parameters could be co-registered as proposed in the current study, while the patient is still being investigated in the cath lab.

Plaque vulnerability and plaque mechanics can be assessed with fluid–structure interaction (FSI). Although FSI would yield added value to the analysis, it is also computationally extremely expensive. Moreover, it was demonstrated that cap thickness is the most important determinant of peak cap stress and can therefore be regarded as a geometrical surrogate marker for peak cap stress [1, 19, 24]. For these reasons, FSI analyses were not performed in this study, as cap thickness was used as an imaging biomarker reflecting the short-term rupture risk.

Between the two side branches used as landmark for the registration procedure, the stack of OCT frames was linearly rotated to match the orientation of the 3D volume. Due to the absence of landmarks between these two side branches, it was not possible to assess the accuracy of this interpolation. Another limitation lies in the fact that, although the 3D reconstruction of the vessel was performed using two angiography views in an identical cardiac phase, the OCT-derived information was acquired all along the cardiac cycle and did not systematically match the same cardiac phase. Accordingly, spatial shift could have been introduced between the parameters derived from angiography and OCT. The change in curvature of the artery is likely to induce, up to a certain extent, different WSS values during the cardiac cycle. Additionally, the thickness of the cap may also undergo some variation due to the mechanical forces compressing the tissues. This issue could be addressed with an ECG-triggered acquisition of the OCT pullback, in such a way that the ROI is imaged during the diastolic phase to match the angiography information. The recently introduced heartbeat OCT technique [40] that can scan a complete coronary artery within one cardiac cycle at 3200 frames/s could also be used to reduce such motion and deformation artifacts.

## Conclusion

Two risk factors are assessed in the coronary artery using two different imaging modalities, namely fibrous cap thickness derived from OCT and WSS computed from angiography. These two parameters are complementary as they provide information about plaque geometry and hemodynamics, respectively. The generation of a unique risk index is enabled by co-registration of the two risk markers using anatomical landmarks such as side branches. The proposed framework is a promising approach for online identification of plaque vulnerability in the clinical arena.
